# Fibroblast growth factor-2 regulates human cardiac myofibroblast-mediated extracellular matrix remodeling

**DOI:** 10.1186/s12967-015-0510-4

**Published:** 2015-05-07

**Authors:** Daniyil A Svystonyuk, Janet MC Ngu, Holly EM Mewhort, Brodie D Lipon, Guoqi Teng, David G Guzzardi, Getanshu Malik, Darrell D Belke, Paul WM Fedak

**Affiliations:** Section of Cardiac Surgery, Department of Cardiac Sciences, University of Calgary, Libin Cardiovascular Institute of Alberta, C880, 1403 29 Street NW, Calgary, Alberta T2N 2T9 Canada

**Keywords:** Extracellular matrix, Cardiac myofibroblasts, Fibrosis

## Abstract

**Background:**

Tissue fibrosis and chamber remodeling is a hallmark of the failing heart and the final common pathway for heart failure of diverse etiologies. Sustained elevation of pro-fibrotic cytokine transforming growth factor-beta1 (TGFβ1) induces cardiac myofibroblast-mediated fibrosis and progressive structural tissue remodeling.

**Objectives:**

We examined the effects of low molecular weight fibroblast growth factor (LMW-FGF-2) on human cardiac myofibroblast-mediated extracellular matrix (ECM) dysregulation and remodeling.

**Methods:**

Human cardiac biopsies were obtained during open-heart surgery and myofibroblasts were isolated, passaged, and seeded within type I collagen matrices. To induce myofibroblast activation and ECM remodeling, myofibroblast-seeded collagen gels were exposed to TGFβ1. The extent of ECM contraction, myofibroblast activation, ECM dysregulation, and cell apoptosis was determined in the presence of LMW-FGF-2 and compared to its absence. Using a novel floating nylon-grid supported thin collagen gel culture platform system, myofibroblast activation and local ECM remodeling around isolated single cells was imaged using confocal microscopy and quantified by image analysis.

**Results:**

TGFβ1 induced significant myofibroblast activation and ECM dysregulation as evidenced by collagen gel contraction, structural ECM remodeling, collagen synthesis, ECM degradation, and altered TIMP expression. LMW-FGF-2 significantly attenuated TGFβ1 induced myofibroblast-mediated ECM remodeling. These observations were similar using either ventricular or atrial-derived cardiac myofibroblasts. In addition, for the first time using individual cells, LMW-FGF-2 was observed to attenuate cardiac myofibroblast activation and prevent local cell-mediated ECM perturbations.

**Conclusions:**

LMW-FGF-2 attenuates human cardiac myofibroblast-mediated ECM remodeling and may prevent progressive maladaptive chamber remodeling and tissue fibrosis for patients with diverse structural heart diseases.

## Background

Structural cardiac remodeling with myocardial fibrosis is a hallmark of progression to heart failure [1]. The cardiac fibroblast maintains extracellular matrix (ECM) homeostasis by balancing ECM production and turnover [2]. After myocardial infarction (MI), quiescent fibroblasts are stimulated by the pro-fibrotic cytokine transforming growth factor (TGF)-β1 to become activated myofibroblasts [3-5]. Persistent myofibroblast activation leads to ECM dysregulation with excessive ECM turnover resulting in net ECM deposition and tissue fibrosis [6]. Myocardial fibrosis is strongly associated with maladaptive post-MI structural remodeling and clinical decompensation [7]. Clinical therapies aimed at preventing persistent myofibroblast activation in the post-MI heart may attenuate maladaptive cardiac remodeling and improve clinical outcomes [6,5].

Contemporary approaches to the surgical treatment of the failing heart do not directly target myofibroblast activation and fibrosis. Novel “biosurgical” approaches are emerging such as injectable protein, gene, and cell therapies that may be capable of directly targeting such processes [8-11]. Identifying and validating key bioactive proteins is critical to the development of these novel approaches. Specific bioactive proteins play an important role in maintaining ECM homeostasis by regulating the interaction between fibroblasts and their adjacent collagen matrix [12]. We believe that such bioactive proteins could be leveraged as effective therapeutic targets for patients at risk of cardiac remodeling and heart failure. We previously showed that surgical cell transplantation induced significant fibroblast growth factor (FGF)-2 expression in the post-MI myocardium that limited post-MI maladaptive structural cardiac remodeling [10]. We also found FGF-2 to be an effective anti-remodeling therapy when used in a biosurgical approach with a low-molecular weight FGF-2 (LMW-FGF-2) enhanced biomaterial surgically implanted on the epicardial surface of the heart after MI [11]. These data and others suggest that LMW-FGF-2, through its bioactive effects on ECM homeostasis, may be a promising target as an anti-remodeling strategy after MI.

LMW-FGF-2 is a multifunctional biopeptide known to regulate numerous cellular processes that serve to maintain myocardial structure and function [13]. However, LMW-FGF-2 is most recognized for its influence as a potent angiogenic agent. The therapeutic application of FGF-2 for cardiac ischemia to stimulate angiogenesis was found to be safe but not clinically effective [14]. Interestingly, studies show that endogenous LMW-FGF-2 may provide important cardioprotective effects in ischemic hearts without improving angiogenesis or tissue perfusion [15,16]. These data may indicate that LMW-FGF-2 could be best leveraged as an effective anti-fibrotic therapy. However, the direct influence of LMW-FGF-2 on cardiac myofibroblast-mediated fibrosis is not well understood. In this study, for the first time, we provide a novel assessment of the effects of LMW-FGF-2 on human cardiac myofibroblast-mediated ECM remodeling using an innovative 3-dimensional (3D) *in vitro* model.

## Methods

### Human cardiac myofibroblast isolation and expansion

Right atrial appendage and left ventricular apical myocardial biopsies were obtained from consenting male and female patients undergoing cardiac surgery using cardiopulmonary bypass at Foothills Medical Center (Calgary, Alberta). All experiments involving human tissue were approved by Conjoint Health Research Ethics Board at the University of Calgary and conform to the Declaration of Helsinki. Samples were minced and dissociated in 0.2% Collagenase Type II at 37°C in an Isotemp® Dry Bath (Fisher Scientific) with gentle stirring. Myofibroblast cell suspension was collected and remnant tissue was removed using a tissue strainer of 40 μm pore size (BD Falcon™). Collected cells were centrifuged and the cell pellet was subsequently seeded in complete medium composed of Iscove’s Modified Dulbecco’s Medium (IMDM) supplemented with 10% fetal bovine serum plus 50,000 units of penicillin and 50,000 μg of streptomycin. Cells were cultured at 37°C with a 5% CO_2_ atmosphere. Cells from passage 4–8 were used for these experiments. The morphology of the cultured cells was consistent with myofibroblasts as examined using phase-contrast light microscopy. To further characterize the cells, immunocytochemistry was performed to confirm the presence of several fibroblast-specific markers: fibronectin, vimentin, fibroblast surface protein and discoidin domain receptor-2. Greater than 95% of the cultured cells from passage 4 stained positive for specific fibroblast markers as previously described by our group [17].

### Assessment of 3D collagen ECM remodeling

Cultured human cardiac myofibroblasts from passage 4–8 were serum-starved for 24 hours. Each experiment utilized cells from the same passage. Myofibroblasts were trypsinized and added to a liquid form of neutralized rat-tail type I collagen (1.8 mg/mL, BD Biosciences) at high density (2.5 × 10^5^ cells/mL). Solutions were incubated at 37°C to allow for gel polymerization. Immediately after polymerization, 500 μL of IMDM either alone (serum-free medium [SFM]) or containing 10 ng/mL human recombinant TGF-β1 (Gibco-Invitrogen, Frederick MD), with or without 20 ng/mL LMW-FGF-2 (Invitrogen, Camarillo, CA, USA) was added to the culture wells and plates were further incubated overnight. To initiate ECM contraction, the cell-ECM constructs were released from the well wall using a sterile micro-spatula (Corning®). Serial images of the ECM dimensions were obtained from the time of release (baseline) and at 24 hours. ImageJ analysis software (NIH, USA) was used to measure the area of ECM contraction as a quantitative measure of ECM remodeling.

### Assessment of myofibroblast activation and ECM remodeling in individual cells

We adapted a novel cell culture platform from Mohammadi and co-workers that employs a floating thin collagen gel supported by rigid nylon grids to isolated single cells within 3D collagen matrices [18]. Individual myofibroblasts were assessed for cell activation (by morphology) and their local effects on adjacent ECM remodeling by image analysis. In brief, nylon screens with 200 × 200 μm openings (Dynamic Aqua Supply, Surrey, BC) were cut into 2 × 2 cm squares. Type I bovine dermal collagen (5.9 mg/mL, Advanced Biomatrix, San Diego, CA) was diluted to a working concentration of 1.0 mg/mL and neutralized to a pH = ~7.4 using 0.1 N NaOH. A 100 μL volume of the collagen solution was poured onto a parafilm-coated culture dish (hydrophobic surface). Nylon squares were placed carefully on top of the collagen, allowing for each grid opening to be filled with the solution and incubated to ensure polymerization of the collagen gel. Resultant collagen-coated constructs were gently detached from the culture dish using warm PBS, inverted and floated in serum-free IMDM culture medium ±10 ng/mL human recombinant TGFβ1, with or without 20 ng/mL LMW-FGF-2. Human cardiac myofibroblasts from the same passage were seeded at low density (1 × 10^4^ cells) allowing single cells in each grid space. Treatments were maintained for 24 hours.

### Immunocytochemistry and confocal microscopy

After functional remodeling data were collected, the cell-ECM constructs were immediately fixed in 4% PFA at RT for 30 minutes. Constructs were then simultaneously blocked and permeabilized at RT for 1 hour by incubating in blocking buffer containing 0.1% Triton® X-100. Constructs were incubated in primary antibody (mouse anti-α-SMA at 1:500, Sigma-Aldrich) for 48 hours, washed and incubated in secondary antibody (Alexa Fluor® 488 goat anti-mouse at 1:500, Invitrogen) at RT for 1 hour with gentle shaking. Lastly, constructs mounted onto microscope glass slides in Prolong® Gold Antifade Reagent containing DAPI for nuclear visualization. All fluorescent images were captured using confocal laser microscopy (LSM 5, Carl Zeiss) and processed using Zen software. Cells positive for α-SMA were counted and divided by the total DAPI (nuclei) for each image. α-SMA-positive cells of 8 random images from each group were averaged and expressed as a percentage (%) of the total cell number.

### Collagen synthesis by [^3^H]-proline incorporation

To measure collagen synthesis, 1 μCi/mL [^3^H]-proline was added to the supernatant treatment solution of the collagen gels containing human cardiac myofibroblasts from the same passage. After the functional ECM remodeling data was collected (48 hrs.), the collagen gels were centrifuged and the extracellular proline was removed by aspiration of the supernatant. Cells were lysed and residual [^3^H]-proline in the collagen gels was removed by incubating the cells for 5 minutes at room temperature in 1 mL of a solution containing: 50 mM Tris–HCl (pH 7.4), 150 mM NaCl, % Triton x-100, 0.5 % Sodium deoxycholate, 0.04% beta mercapto-ethanol, 1 mM EDTA and centrifuging as above. The collagen gels were dissolved in 0.4 N NaOH, and an aliquot of final solution was added to scintillation vials containing 5 ml of Ecolite scintillation cocktail to determine [^3^H]-proline incorporated into protein (largely collagen). Protein concentration was measured using the Bradford assay. Nonspecific incorporation of [^3^H]-proline was defined as that observed in the presence of an excess of unlabeled proline; or in collagen gels formed without myofibroblasts.

### In situ zymography

*In situ* zymography was modified for use in the collagen gel contraction assay as we previously described [17]. In brief, constructs containing human cardiac myofibroblasts from the same passage were prepared as described in the previous section. DQT™ Gelatin-FITC (Molecular Probes), which emits a quantifiable green fluorescent signal when cleaved by proteases, was added to the liquid gel solution at a final concentration of 10 μg/mL prior to gel polymerization. Subsequently, constructs were fixed in 4% PFA for 30 minutes at RT, washed with PBS, and mounted on microscope glass slides with Prolong Gold Antifade Reagent. Z-stack images from 8 random fields of each collagen gel were captured using confocal laser microscopy (LSM 5, Carl Zeiss) and images were analyzed using Volocity software (PerkinElmer). Bright green fluorescent spots indicating proteolytic digestion in the acquired 3D gel image were identified as “green fluorescent objects” on the Volocity software platform and the total volume of these objects was summed as “Total Volume of Green Fluorescence”. Mean fluorescence intensity of each analyzed 3D gel image was generated automatically by the Volocity software, by averaging the fluorescence intensity of all the “green fluorescent objects” identified in the image. The total green fluorescence activity, known to be proportionate to the total protease activity, was calculated by multiplying the total volume of green fluorescence of each 3D image by its mean fluorescence intensity. The total protease activity in collagen gel normalized to the corresponding 3D gel image volume to allow for comparison between images:

$$ \frac{\mathrm{Total}\kern0.5em \mathrm{Protease}}{\mathrm{Activity}}=\frac{\mathrm{Mean}\kern0.5em \mathrm{Fluorescence}\kern0.5em \mathrm{Intensity}\times \mathrm{Total}\kern0.5em \mathrm{Volume}\kern0.5em \mathrm{of}\kern0.5em \mathrm{Green}\kern0.5em \mathrm{Fluorescence}}{\mathrm{Total}\kern0.5em \mathrm{Image}\kern0.5em \mathrm{Volume}} $$

### Extraction of cells from ECM constructs

Cardiac myofibroblasts were harvested from the cell-ECM constructs after the functional remodeling data were collected. Cells were released by incubating the constructs in 0.2% Collagenase Type II with constant agitation until completely dissolved. The cell suspension was centrifuged and the supernatant was discarded leaving the cell pellet for further testing as described.

### Flow cytometry

Cell pellets harvested from each collagen gel were resuspended in polystyrene round bottom 12 × 75 mm^2^ Falcon tubes. Cells were fixed in ice-cold methanol at −20°C, followed by washing with PBS + 1% bovine serum albumin (BSA). Cells were permeabilized by incubation with PBS + 1% Triton-X and then collected by centrifugation. Cells were stained with anti-α-SMA conjugated with FITC (1:200 dilution; Sigma-Aldrich) for flow cytometry analysis.

### Cell apoptosis

Harvested cells pellets were resuspended in 1× Annexin V binding buffer (BD Biosciences, Mississauga, ON) with propidium iodide (1:100 dilution, BD Biosciences) and FITC Annexin V (1:100 dilution, BD Biosciences) before flow cytometry analysis.

### Quantitative RT-PCR

Real-time PCR was performed on mRNA isolated, using a Qiagen RNeasy extraction kit, from cardiac myofibroblasts extracted from the collagen gels. mRNA was converted to cDNA for RT-PCR using Quantitect reverse transcription kit (Qiagen, CA, USA). TIMP-1 and TIMP-2 mRNA was quantified using human primer pairs, TIMP-1 (forward: aattccgacctcgtcatcag; reverse: tgcagttttccagcaatgag) & TIMP-2 (forward: tgatccacacacgttggtct; reverse: tttgagttgcttgcaggatg), (University of Calgary, DNA Lab Core Facility) and a Quantitect SYBR Green PCR kit (Qiagen, CA, USA) and Bio-Rad Icycler. Gene expression levels were measured against human 18 s RNA (Qiagen QuantiTect primer QT00199367) as a housekeeping gene.

### Luminex multiplex assay

Immediately following functional data collection within the collagen gel contraction assay 50 μL of culture media was collected from each well. The concentration of human MMPs and TIMPs within the culture media was measured using Luminex multiplex analysis (Eve Technologies, Calgary, AB, Canada).

### Assessment of cell morphology

Cells were fixed in 4% PFA and permeabilized in 0.1% Triton-X. Myofibroblast actin cytoskeleton was marked with Alexa Fluor 488 phalloidin (Life Technologies, Burlington, ON) while cell nuclei were stained with DAPI. Collagen fibers were imaged using confocal reflectance microscopy (CRM) with a confocal laser microscope (LSM 5, Carl Zeiss) as previously described by our group [17]. Morphological parameters included cell extension length and roundness (a dimensionless shape factor). Cell extension length was measured from the center of the cell to the tip of an extension using ImageJ software (version 1.48, NIH, USA). Measurement of roundness was performed using the Multi-Cell Outliner plug-in for ImageJ (http://rsbweb.nih.gov/ij/plugins/multi-cell-outliner.html). In brief, values approaching 1 are representative of a round cell morphology while values approaching 0 are considered more non-round [19].

### Quantification of cell-mediated ECM remodeling

Assessment of collagen fiber alignment was performed as described by Mohammadi and co-workers [18]. Collagen fibers were imaged using confocal reflectance microscopy (CRM) with a confocal laser microscope (LSM 5, Carl Zeiss) as previously described by our group [17]. In brief, the Fast Fourier Transform (FFT) function of ImageJ was used to quantify the distribution of collagen fibers at a given angle from the acquired images. FFT produces a spectral image in the frequency domain of and orthogonal to the original intensity image [20]. To quantify the directionality of the original image, pixel intensities were summed along a straight line from the image center at different angle intervals using the Oval Profile ImageJ plug-in (http://rsb.info.nih.gov/ij/plugins/oval-profile.html). An image in which the collagen fibers are randomly distributed display relatively constant pixel intensities at different angles. Orientation of collagen fibers in a specific direction would correspond to higher pixel intensities at that particular angle. From the resultant intensity curve, we define a collagen fiber alignment index value by calculating the area under the curve bound by the peak intensity value ± 10°.

### Statistical analysis

All group data are presented as mean ± SD. Data were obtained from a representative experiment of which each was repeated in triplicate. When only two groups were compared, a Student’s *t*-test was performed. For comparison of more than two groups, one-way ANOVA was used and followed by appropriate *post hoc* comparison tests. All statistical analyses were performed using GraphPad Prism 6.0, with *p* < 0.05 considered statistically significant.

## Results

### LMW-FGF-2 prevents human cardiac myofibroblast-mediated ECM remodeling

**Figure 1:**
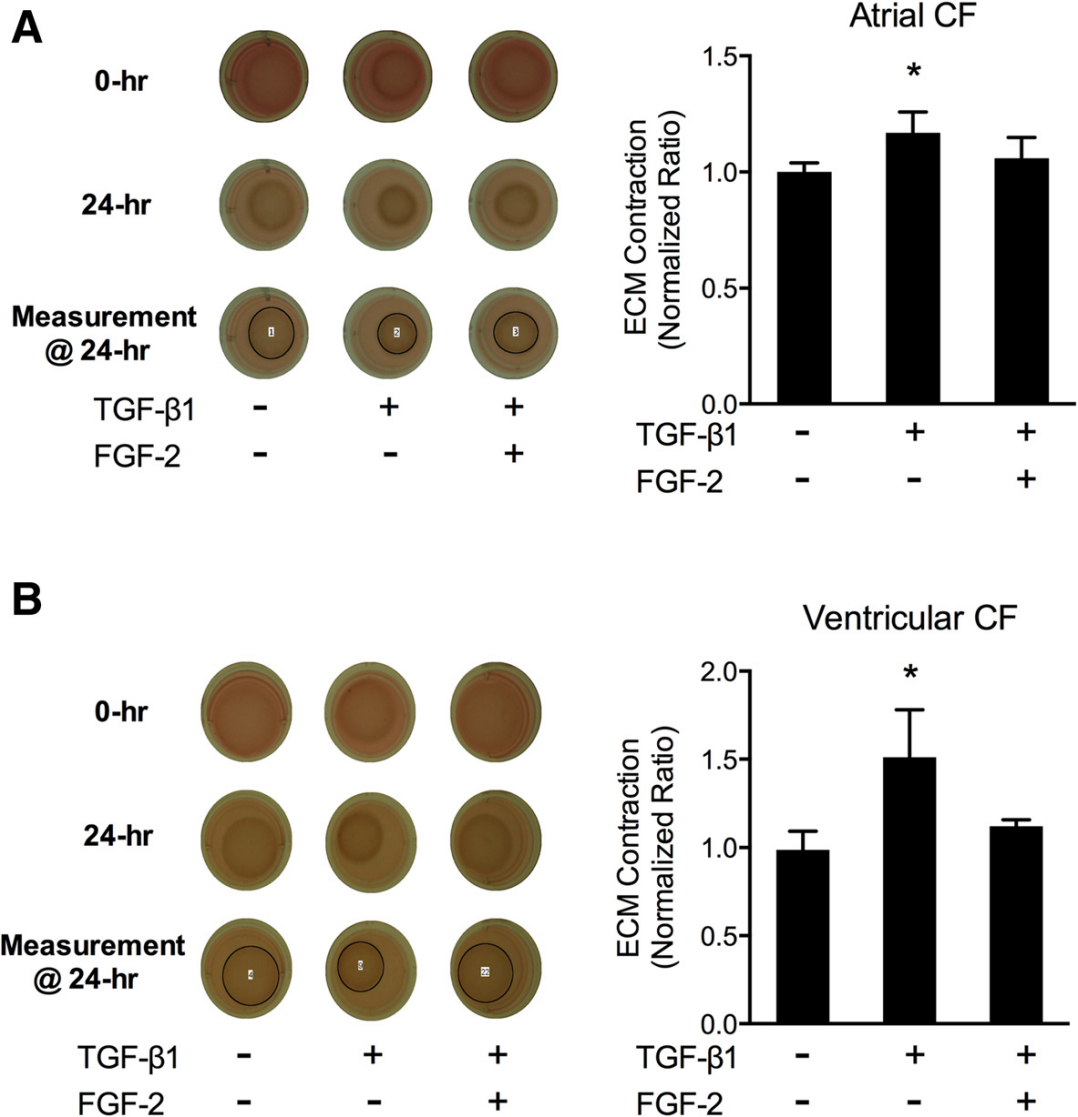
Extracellular matrix remodeling by human cardiac myofibroblasts. TGFβ1 stimulates both atrial **(A)** and ventricular **(B)** myofibroblast-mediated collagen gel contraction as compared to controls. This effect was inhibited in the presence of LMW-FGF-2. Data presented were obtained from multiple individual experiments (4 experiments, total N = 14 per group for A; 3 experiments, total N = 8 per group for B) and all values were normalized to the corresponding control groups. Bars represent mean ± SD; *p < 0.01, **p < 0.001; abbreviation: CF – cardiac fibroblasts.

Cardiac myofibroblasts *in vitro* will remodel and contract collagen matrices in proportion to the extent of their activation [21,22]. TGFβ1, a critical pro-fibrotic mediator of human diseases, stimulates cardiac fibroblasts to undergo phenotypic conversion into active myofibroblasts resulting in collagen ECM remodeling and fibrosis [4]. Accordingly, we examined the effects of exogenous LMW-FGF-2 on TGFβ1-induced human cardiac myofibroblast-mediated collagen remodeling (Figure 1). LMW-FGF-2 exposure completely prevented TGFβ1-stimulated human cardiac myofibroblast ECM remodeling as reflected by the attenuation of TGFβ1-stimulated ECM contraction. Importantly, both atrial and ventricular human cardiac myofibroblasts stimulated with TGFβ1 demonstrated similar reductions in ECM contraction in the presence of LMW-FGF-2.

### LMW-FGF-2 exposure does not induce apoptosis

To confirm that the anti-fibrotic effect of LMW-FGF-2 is not a result of cell loss due to apoptosis, Annexin V staining was performed for detection of externalized apoptotic markers (phosphatidylserine). LMW-FGF-2 exposure did not induce apoptosis relative to TGFβ1 without LMW-FGF-2 exposure (1.10 ± 0.62% vs. 1.03 ± 0.61%, n = 3, p = ns), suggesting that the observed inhibitory effects on collagen gel contraction were not a consequence of cell apoptosis (data not shown in Figures).

### LMW-FGF-2 inhibits TGFβ1-mediated myofibroblast activation

**Figure 2:**
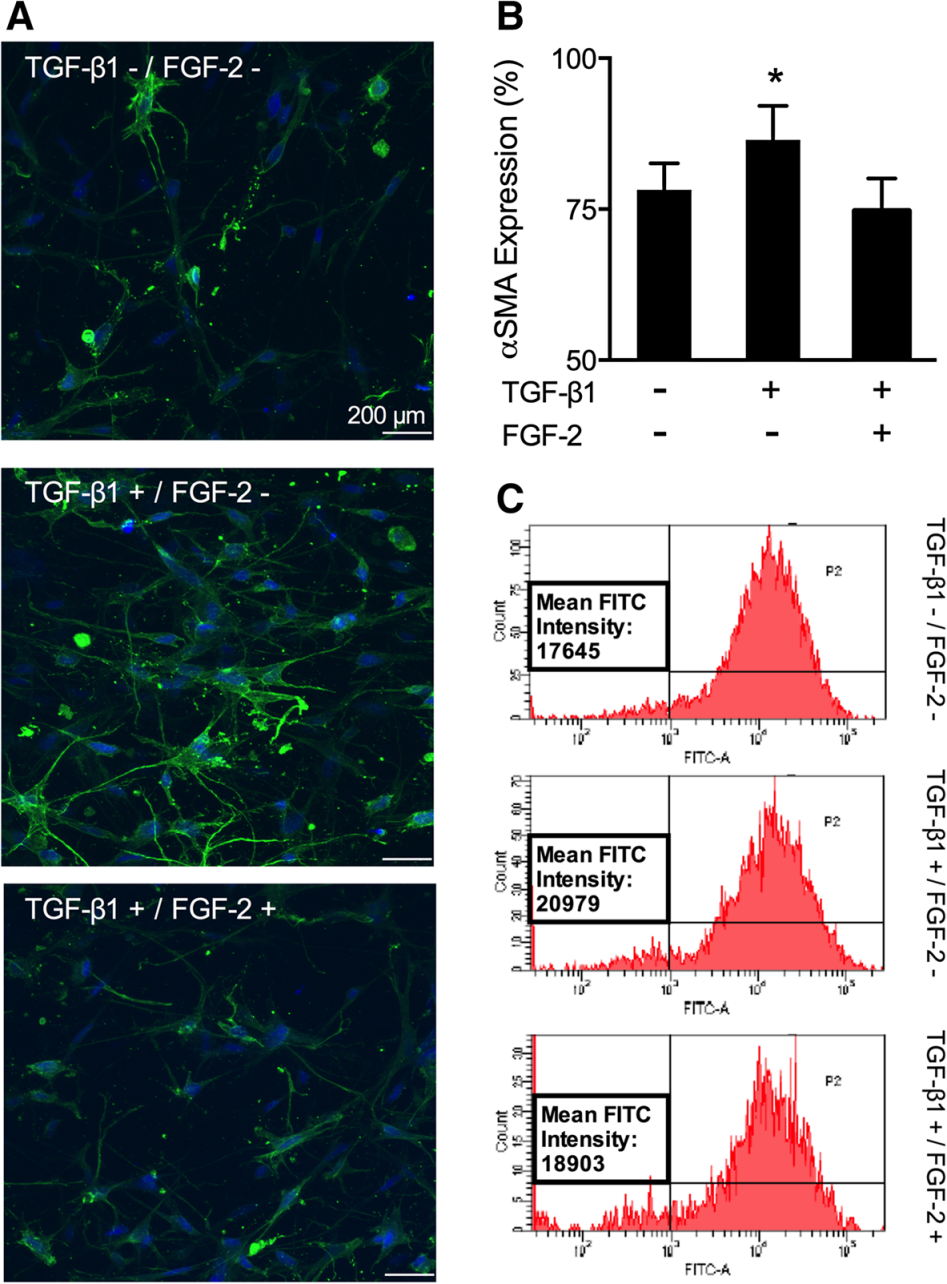
Effects of LMW-FGF-2 on myofibroblast activation. **(A)** Representative confocal microscopic images of cardiac myofibroblasts embedded in a collagen gel, following treatments (control, TGFβ1 and LMW-FGF-2 + TGFβ1). Cells were stained for α-SMA (green) and nuclei (DAPI; blue). **(B)** TGFβ1 increased α-SMA expression while LMW-FGF-2 diminished α-SMA expression**.** Bars represent mean ± SD; *p < 0.01. **(C)** α-SMA was labeled with FITC-conjugated antibody and the intensity of a-SMA expression was measured using flow cytometry. TGFβ1 increased the cellular expression of α-SMA and this effect was abolished by LMW-FGF-2.

The activation of myofibroblasts within ECM constructs was assessed by immunostaining for α-SMA (Figure 2A) with quantification by image analysis (Figure 2B). TGFβ1 stimulated α-SMA expression in human cardiac myofibroblasts within 3D collagen ECM constructs. The addition of LMW-FGF-2 prevented α-SMA expression increases with TGFβ1 exposure. Changes in α-SMA expression were also confirmed by flow cytometry using cells extracted from the ECM constructs after ECM remodeling was initiated (Figure 2C). These data suggest that LMW-FGF-2 prevented myofibroblast activation in response to TGFβ1 exposure.

### LMW-FGF-2 attenuates TGFβ1-induced ECM dysregulation

**Figure 3:**
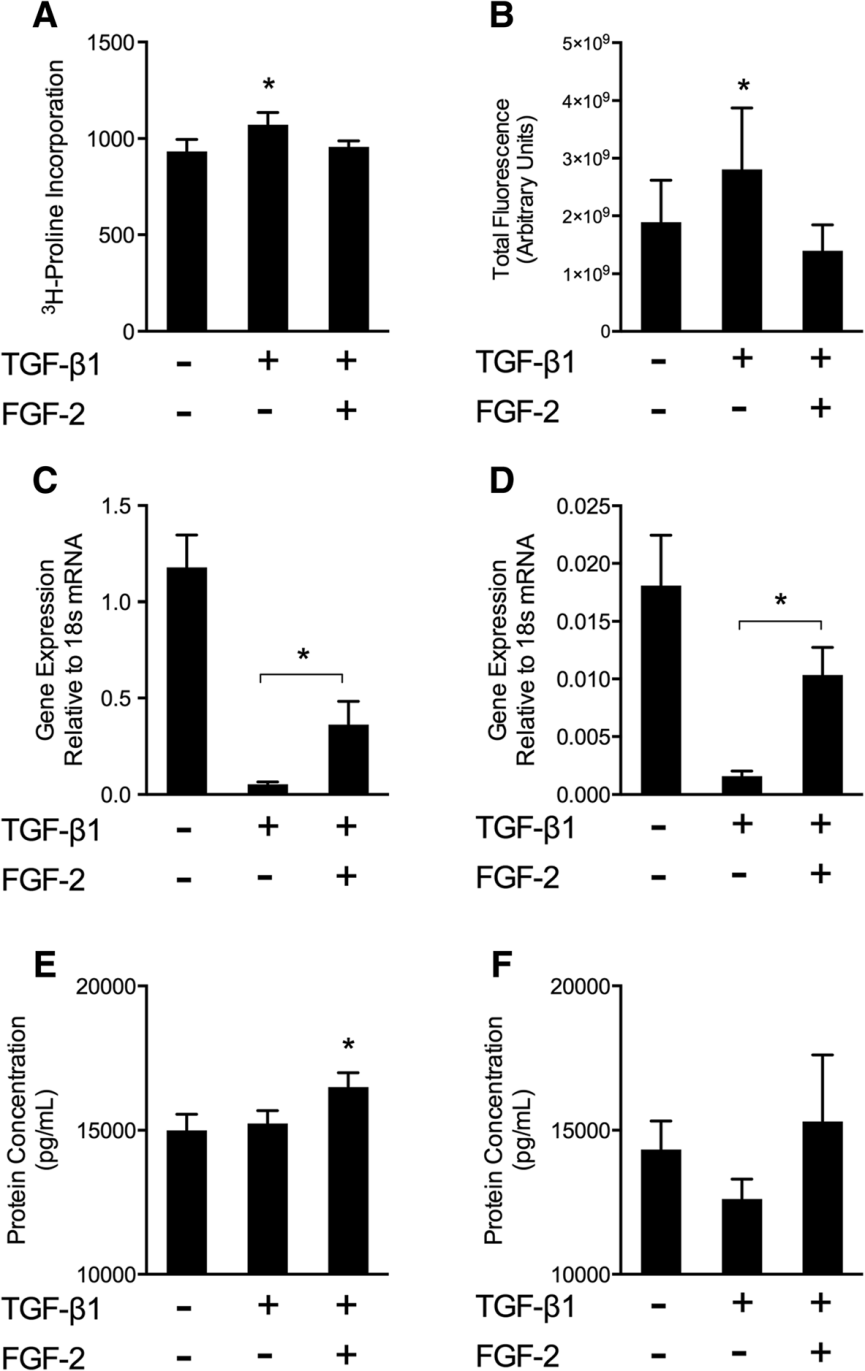
ECM regulation by collagen synthesis, protease activity, and TIMP inhibition. **(A)** Collagen synthesis was assessed by measurement of ^3^H-proline incorporation. TGFβ1 increased ^3^H-proline incorporation. This effect was abolished by addition of LMW-FGF-2. **(B)** ECM degradation was assessed by *in situ* zymography using embedded DQ™ Gelatin-FITC to measure matrix proteolysis. Total protease activity was quantified as total fluorescent signal per image volume. TGFβ1 increased total protease activity while LMW-FGF-2 attenuated this response. **(C** and **D)** Expression of TIMP-1 and TIMP-2, respectively, at the mRNA levels are presented. TGFβ1 significantly reduced both TIMP-1 and TIMP-2 expression while LMW-FGF-2 partially restored expression. **(E)** At the protein level, TGFβ1 did not alter TIMP-1, however the presence of LMW-FGF-2 resulted in increased TIMP-1 protein. **(F)** Though the results for TIMP-2 were not statistically significant, a trend toward decreased TIMP-2 protein was noted in the TGFβ1 group and restored in the presence of LMW-FGF-2. *, p < 0.05.

ECM homeostasis requires a balance between ECM deposition (synthesis) and ongoing degradation. Collagen synthesis was assessed by [^3^H]-proline incorporation. The pro-fibrotic peptide TGFβ1 induced a significant increase in collagen synthesis, which was observed to decrease toward baseline in the presence of LMW-FGF-2 (Figure 3A). Excessive ECM turnover (dysregulation) results from imbalances between matrix protease activities and their endogenous inhibitors, TIMPs. *In situ* zymography was used to directly assess regional ECM protease activity within the collagen gel microenvironment (Figure 3B). Indicative of ECM dysregulation and a propensity for structural remodeling, TGFβ1 increased total protease activity within cell-ECM constructs. LMW-FGF-2 abolished the TGFβ1-associated increases in protease activity. MMP concentrations in the conditioned media of the TGF-β1 and LMW-FGF-2 groups (MMP-1, MMP-2, MMP-7, MMP-9) were not significantly different (data not shown). However, TGFβ1 profoundly inhibited TIMP expression at both the mRNA and protein levels (Figures 3C-F) thereby increasing the capacity for ECM turnover by reducing protease inhibition. TIMP expression was partially restored to baseline by the addition of LMW-FGF-2. These data collectively indicate that LMW-FGF-2 attenuates TGFβ1-induced ECM dysregulation.

### LMW-FGF-2 attenuates TGFβ1-induced myofibroblast morphologic changes

**Figure 4:**
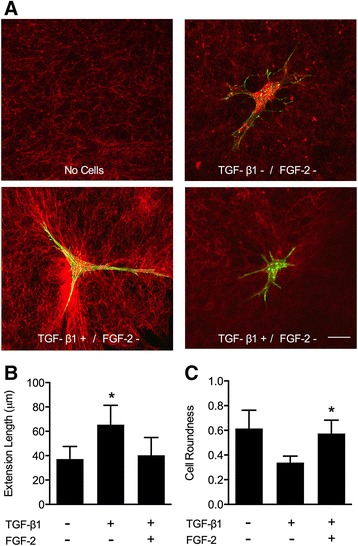
Myofibroblast morphology: extension length and roundness. **(A)** Representative confocal images of single isolated human cardiac myofibroblasts embedded into a thin collagen nylon-based gel. Following treatments (control, TGFβ1, TGFβ1 + LMW-FGF-2), cells were stained for F-actin (phalloidin; green) while adjacent collagen matrix was visualized using confocal reflectance (red). **(B)** Cell extension length was measured from the cell centroid to the tip of a given cell extension. TGFβ1 increased mean cell extension length while LMW-FGF-2 restored levels close to baseline. Bars represent mean ± SD; *p < 0.05. **(C)** Roundness, a dimensionless shape factor, was quantified for each treatment group. Values closer to 1.0 represent round phenotypes while decreasing values <1.0 are representative of a star-shaped phenotype (activated). TGFβ1 treatment favored a more stellate-cell shape while LMW-FGF-2 showed morphology consistent with non-activated cells. Bars represent mean ± SD; *p < 0.05. Scale bar = 20 μm.

To better understand the role of LMW-FGF-2 on myofibroblast activation and cell-induced remodeling, a novel nylon-based thin fixed collagen grid was used to study isolated cells and their local surrounding collagen matrix. Unlike the previously described collagen gel contraction model, these thin gels permit assessments of altered cell morphology and regional matrix deformations without concomitant changes in the collagen network surface area (compaction). Validated morphological assessments were utilized to study the extent of myofibroblast activation by cytoplasmic extension length and roundness [18]. TGFβ1 stimulated myofibroblasts were observed to have longer cell extensions and a more stellate morphology characteristic of activated myofibroblasts (Figure 4). In contrast, LMW-FGF-2 treatment resulted in shorter cell extensions and a round cell shape consistent with control myofibroblast morphology (Figure 4E and F). Collectively, these data suggest LMW-FGF-2 prevents activation of myofibroblasts.

### LMW-FGF-2 attenuates TGF-β1 induced cardiac myofibroblast ECM remodeling

**Figure 5:**
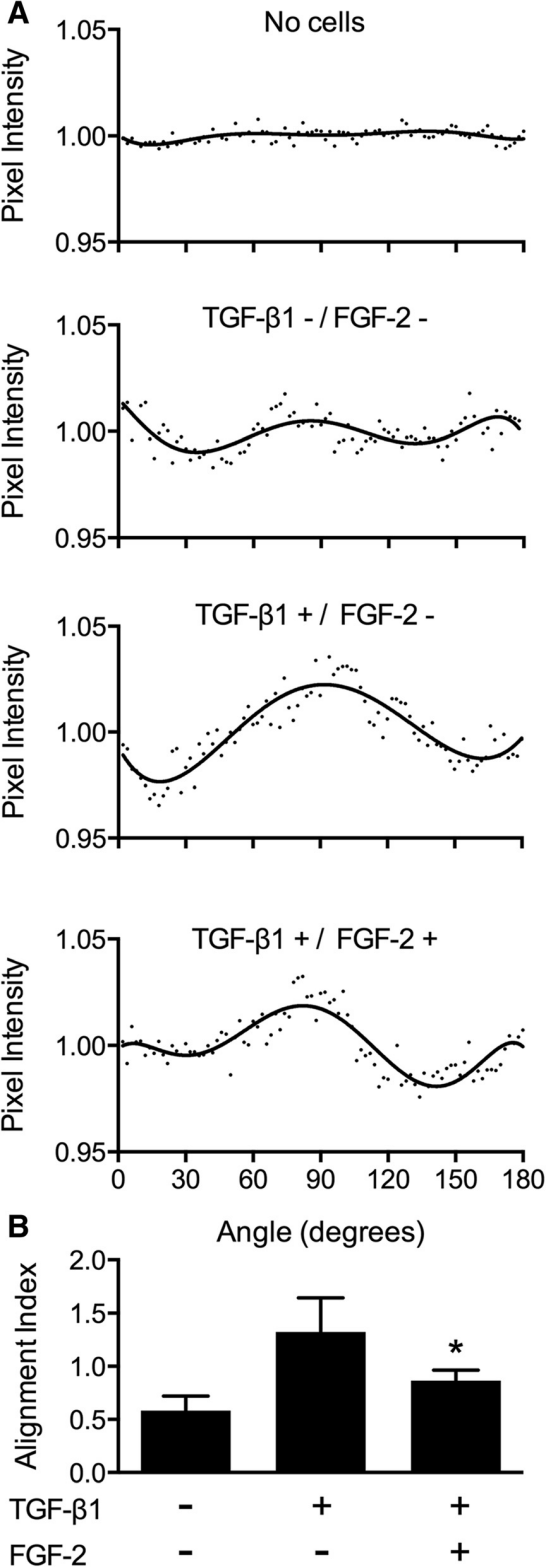
Extracellular matrix remodeling: collagen fiber alignment. Collagen fiber alignment was assessed parallel to cell extensions as a measure of cell-mediated ECM remodeling. **(A)** Normalized pixel intensity curves generated from FFT analysis for each treatment group relative to a no-cell control. Randomly oriented collagen fibers result in constant pixel intensity at any given angle. Alignment of fibers parallel to cell extensions result in increased intensity at certain angles. **(B)** Alignment index is calculated by taking the area under the curve ± 10 degrees from the maximum intensity value. TGFβ1 showed the greatest collagen fiber alignment while LMW-FGF-2 attenuated this response. Bars represent mean ± SD; *p < 0.05.

The cell-mediated remodeling of the local ECM was assessed as a function of collagen fiber alignment at the tips of cell extensions, as described by Mohammadi and colleagues [18]. Collagen fibers preferentially align in the direction of the underlying cell-induced strain field [23]. We assume that the extent of collagen fiber alignment is relative to the strength of the strain field, which is a reflection of the magnitude of remodeling. Confocal reflectance microscopy was used to document changes in matrix organization among the different treatment groups. The collagen matrices in the absence of cells consist of randomly oriented fibers with no directional preference (Figure 4A). In contrast, the presence of inactive myofibroblasts resulted in their partial orientation, suggesting minor ECM remodeling (Figure 4B). Relative to these controls, we observed significant ECM remodeling following myofibroblast activation with TGFβ1 that was lessened with LMW-FGF-2 treatment (Figure 4C and D). These observations were simultaneously confirmed through Fast Fourier Transform (FFT) analysis, showing that LMW-FGF-2 prevented the effects of TGFβ1 such that the alignment index values approached baseline levels (Figure 5A and B). These novel data confirm for the first time that LMW-FGF-2 attenuates TGFβ1-induced human myofibroblast-mediated ECM remodeling.

## Discussion

Under pathological conditions, elevated levels of TGFβ1 have been well studied in stimulating the fibroblast-to-myofibroblast transition [24]. Sustained myocardial TGFβ1 activity in the post-MI heart drives persistent myofibroblast activation and associated ECM dysregulation resulting in maladaptive structural remodeling [4]. This study examined and validated the influence of LMW-FGF-2 on human cardiac myofibroblast-mediated ECM remodeling. Using a novel *in vitro* 3D model, we validate for the first time that LMW-FGF-2 can prevent TGFβ1-induced myofibroblast-mediated 3D extracellular matrix remodeling in cells from human cardiac tissue. Our data show that LMW-FGF-2 attenuated TGFβ1-induced changes in α-SMA expression, collagen synthesis, and TIMP expression. These alterations collectively reduced ECM turnover and prevented net collagen accumulation (fibrosis) resulting in preserved ECM architecture.

Fibrosis is a complex phenomenon. The role of LMW-FGF-2 in mediating cardiac ECM regulation and fibrosis pathways is unclear. Selected prior studies suggest that cardiac FGF-2 expression is pro-fibrotic. Li and colleagues documented elevated FGF-2 in fibrotic human atria inferring a pro-fibrotic causative role for FGF-2 [25]. In addition, Virag and co-workers detected greater infarct scar formation after cardiac-specific over-expression of FGF-2 in post-MI mutant mice [16]. In contrast to these prior *in vivo* descriptive studies, our data indicates a profound anti-fibrotic effect of LMW-FGF-2 on human cardiac myofibroblasts within a 3D collagen matrix. Our findings are consistent with the work of others who observed that FGF-2 regulates TGFβ1-mediated myofibroblast activity in non-cardiac derived cells [26-29]. Furthermore, and in support of our findings, Suzuki and co-workers documented an anti-fibrotic effect with addition of exogenous FGF-2 to the hearts of hypertensive rats [30]. To explain a possible mechanism of action, Cushing and co-workers showed that FGF-2 stimulates MAPK signaling and blocks the localization of SMADs into the nucleus. The suppression of SMADs normally upregulated by TGFβ1 prevented the activation of genes that induce myofibroblast activity and differentiation [31]. Although unproven, the observed effects on ECM regulation (collagen synthesis, degradation and TIMP expression) may be secondary events to the suppression of myofibroblast activity by LMW-FGF-2 exposure.

The molecular and cellular pathways that underlie cardiac fibrosis and remodeling are complex and synergistic. However, human 3D cell culture models have a number of distinct advantages over traditional cell culture experiments that may allow for improved translation to the human condition. First, we used cardiac myofibroblasts from human atria and ventricles undergoing heart surgery. While we recognize that differences in patient characteristics may contribute to myofibroblast heterogeneity, the capacity to induce expected changes through our treatment groups was preserved. Second, we utilized an ECM composed of type I collagen which is the primary isoform in the human heart [32]. Third, we embedded the cells in 3D matrix constructs. 3D matrices best mimic *in vivo* tissue microenvironments and permit normal physiologic cell-ECM interactions [33]. Leveraging this knowledge, we confirmed effects of LMW-FGF-2 on human cardiac myofibroblast-mediated ECM remodeling using a novel cell culture platform. This model may better reflect *in vivo* physiologic conditions on account of its floating gel system with rigid lateral boundaries that may mimic basement membranes [18].

Our recent studies have implicated LMW-FGF-2 as a key paracrine mediator underlying the observed benefits of cell transplantation in attenuating structural cardiac remodeling [34,10,9]. Recently, we leveraged this cardioprotective capacity using a unique LMW-FGF-2-enhanced biomaterial and found significant benefits on preventing early post-MI maladaptive remodeling and fibrosis [11]. While this biomaterial showed favorable effects on the physiology of the post-MI heart, the effects on endogenous cell populations remained poorly understood. Spinale and co-workers recently examined the effects of myocardial injections of biocomposite materials on myofibroblast activity in relation to LV remodeling post-MI [8]. Growth factors and biomaterials are gaining popularity over cell therapy approaches due to the barriers to clinical translation with cell-based regenerative approaches. Our data support the use of LMW-FGF-2 to directly target myofibroblast-mediated ECM remodeling in the failing heart.

We believe that the present study substantiates the anti-fibrotic influence of LMW-FGF-2 on human cardiac myofibroblasts and demonstrates a proof-of-concept for using LMW-FGF-2 to specifically target ECM dysregulation in the post-MI human heart. When employed as a clinical therapy to induce angiogenesis, Ruel and Sellke showed that FGF-2 delivery via sustained-release capsules was proven to be safe for patients and effective at increasing myocardial revascularization [35]. We speculate that LMW-FGF-2 is both safe and effective when administered in patients at the time of post-MI remodeling when TGFβ1 levels are increased, myofibroblasts are activated, and ECM homeostasis is disturbed toward myocardial fibrosis. Differences in fibrotic capacity have been observed between atrial as compared to ventricular cardiac myofibroblasts [36]. We confirmed an anti-fibrotic response of LMW-FGF-2 on both atrial and ventricular derived human cardiac myofibroblasts suggesting that LMW-FGF-2 therapy could be effective at limiting structural remodeling in both the atria and ventricles in patients at risk of heart failure.

## Conclusions

We uniquely demonstrated through two novel 3D cell culture platforms the capacity of LMW-FGF-2 to attenuate human cardiac myofibroblast activation and adjacent ECM remodeling. This data may provide validation for the use of LMW-FGF-2 in biosurgical approaches that may prevent progressive maladaptive chamber remodeling and tissue fibrosis for patients with diverse structural heart diseases.

## Abbreviations

ECM: Extracellular matrix
MI: Myocardial infarction
TGFβ1: Transforming growth factor-beta1
LMW-FGF-2: Low molecular weight basic fibroblast growth factor
α-SMA: Alpha-smooth muscle actin
MMP: Matrix metalloproteinase
TIMP: Tissue inhibitor of metalloproteinases

## References

[1] Fedak PW, Verma S, Weisel RD, Li RK (2005). Cardiac remodeling and failure: from molecules to man (Part I). Cardiovasc Pathol.

[2] Spinale FG (2002). Matrix metalloproteinases: regulation and dysregulation in the failing heart. CircRes.

[3] Biernacka A, Dobaczewski M, Frangogiannis NG (2011). TGF-β signaling in fibrosis. Growth factors.

[4] Dobaczewski M, Chen W, Frangogiannis NG (2011). Transforming growth factor (TGF)-β signaling in cardiac remodeling. J Mol Cell Cardiol.

[5] Leask A (2010). Potential therapeutic targets for cardiac fibrosis: TGFbeta, angiotensin, endothelin, CCN2, and PDGF, partners in fibroblast activation. Circ Res.

[6] Fraccarollo D, Galuppo P, Bauersachs J (2012). Novel therapeutic approaches to post-infarction remodelling. Cardiovasc Res.

[7] Spinale FG (2007). Myocardial matrix remodeling and the matrix metalloproteinases: influence on cardiac form and function. Physiol Rev.

[8] McGarvey JR, Pettaway S, Shuman JA, Novack CP, Zellars KN, Freels PD (2014). Targeted injection of a biocomposite material alters macrophage and fibroblast phenotype and function following myocardial infarction: relation to left ventricular remodeling. J Pharmacol Exp Ther.

[9] Fedak PW (2008). Paracrine effects of cell transplantation: modifying ventricular remodeling in the failing heart. Semin Thorac Cardiovasc Surg.

[10] Fedak PW, Bai L, Turnbull J, Ngu J, Narine K, Duff HJ (2012). Cell therapy limits myofibroblast differentiation and structural cardiac remodeling: basic fibroblast growth factor-mediated paracrine mechanism. Circulation Heart failure.

[11] Mewhort HE, Turnbull JD, Meijndert HC, Ngu JM, Fedak PW (2014). Epicardial infarct repair with basic fibroblast growth factor-enhanced CorMatrix-ECM biomaterial attenuates postischemic cardiac remodeling. J Thorac Cardiovasc Surg.

[12] Frangogiannis NG (2012). Matricellular proteins in cardiac adaptation and disease. Physiol Rev.

[13] Detillieux KA, Sheikh F, Kardami E, Cattini PA (2003). Biological activities of fibroblast growth factor-2 in the adult myocardium. Cardiovasc Res.

[14] Aviles RJ, Annex BH, Lederman RJ (2003). Testing clinical therapeutic angiogenesis using basic fibroblast growth factor (FGF-2). Br J Pharmacol.

[15] House SL, Bolte C, Zhou M, Doetschman T, Klevitsky R, Newman G (2003). Cardiac-specific overexpression of fibroblast growth factor-2 protects against myocardial dysfunction and infarction in a murine model of low-flow ischemia. Circulation.

[16] Virag JA, Rolle ML, Reece J, Hardouin S, Feigl EO, Murry CE (2007). Fibroblast growth factor-2 regulates myocardial infarct repair: effects on cell proliferation, scar contraction, and ventricular function. Am J Pathol.

[17] Ngu JM, Teng G, Meijndert HC, Mewhort HE, Turnbull JD, Stetler-Stevenson WG (2014). Human cardiac fibroblast extracellular matrix remodeling: dual effects of tissue inhibitor of metalloproteinase-2. Cardiovasc Pathol.

[18] Mohammadi H, Janmey PA, McCulloch CA (2014). Lateral boundary mechanosensing by adherent cells in a collagen gel system. Biomaterials.

[19] Lin S, Baye LM, Westfall TA, Slusarski DC (2010). Wnt5b-Ryk pathway provides directional signals to regulate gastrulation movement. Journal of Cell Biology.

[20] Pang Y, Ucuzian AA, Matsumura A, Brey EM, Gassman AA, Husak VA (2009). The temporal and spatial dynamics of microscale collagen scaffold remodeling by smooth muscle cells. Biomaterials.

[21] Lijnen P, Petrov V, Fagard R (2003). Transforming growth factor-beta 1-mediated collagen gel contraction by cardiac fibroblasts. J Renin Angiotensin Aldosterone Syst.

[22] Lijnen P, Petrov V, Rumilla K, Fagard R (2003). Transforming growth factor-beta 1 promotes contraction of collagen gel by cardiac fibroblasts through their differentiation into myofibroblasts. Methods Find Exp Clin Pharmacol.

[23] Driessen NJ, Cox MA, Bouten CV, Baaijens FP (2008). Remodelling of the angular collagen fiber distribution in cardiovascular tissues. Biomech Model Mechanobiol.

[24] Vaughan MB, Howard EW, Tomasek JJ (2000). Transforming growth factor-β1 promotes the morphological and functional differentiation of the myofibroblast. Exp Cell Res.

[25] Li M, Yi X, Ma L, Zhou Y (2013). Hepatocyte growth factor and basic fibroblast growth factor regulate atrial fibrosis in patients with atrial fibrillation and rheumatic heart disease via the mitogen-activated protein kinase signaling pathway. Exp Ther Med.

[26] Tiede S, Ernst N, Bayat A, Paus R, Tronnier V, Zechel C (2009). Basic fibroblast growth factor: a potential new therapeutic tool for the treatment of hypertrophic and keloid scars. Ann Anat.

[27] Narine K, De Wever O, Van Valckenborgh D, Francois K, Bracke M, DeSmet S (2006). Growth factor modulation of fibroblast proliferation, differentiation, and invasion: implications for tissue valve engineering. Tissue Eng.

[28] Schuliga M, Javeed A, Harris T, Xia Y, Qin C, Wang Z (2013). Transforming growth factor-β-induced differentiation of airway smooth muscle cells is inhibited by fibroblast growth factor-2. Am J Respir Cell Mol Biol.

[29] Silverio-Ruiz KG, Martinez AE, Garlet GP, Barbosa CF, Silva JS, Cicarelli RM (2007). Opposite effects of bFGF and TGF-beta on collagen metabolism by human periodontal ligament fibroblasts. Cytokine.

[30] Suzuki T, Akasaka Y, Namiki A, Ito K, Ishikawa Y, Yamazaki J (2008). Basic fibroblast growth factor inhibits ventricular remodeling in Dahl salt-sensitive hypertensive rats. J Hypertens.

[31] Cushing MC, Mariner PD, Liao JT, Sims EA, Anseth KS (2008). Fibroblast growth factor represses Smad-mediated myofibroblast activation in aortic valvular interstitial cells. FASEB J.

[32] Weber K, Sun Y, Tyagi S, Cleutjens J (1994). Collagen network of the myocardium: function, structural remodeling and regulatory mechanisms. J Mol Cell Cardiol.

[33] Grinnell F (2000). Fibroblast-collagen-matrix contraction: growth-factor signalling and mechanical loading. Trends Cell Biol.

[34] Fedak PW, Szmitko PE, Weisel RD, Altamentova SM, Nili N, Ohno N (2005). Cell transplantation preserves matrix homeostasis: a novel paracrine mechanism. J Thorac Cardiovasc Surg..

[35] Ruel M, Laham RJ, Parker JA, Post MJ, Ware JA, Simons M (2002). Long-term effects of surgical angiogenic therapy with fibroblast growth factor 2 protein. J Thorac Cardiovasc Surg.

[36] Burstein B, Libby E, Calderone A, Nattel S (2008). Differential behaviors of atrial versus ventricular fibroblasts: a potential role for platelet-derived growth factor in atrial-ventricular remodeling differences. Circulation.

